# A Case of Previously Undiagnosed Systemic Lupus Erythematosus and *Mycobacterium tuberculosis* Infection Presenting as Diffuse Alveolar Hemorrhage

**DOI:** 10.1155/2023/3686772

**Published:** 2023-01-11

**Authors:** Henna Iqbal, Benny Screws, Muhammad S. Khan

**Affiliations:** ^1^Biomedical Sciences Department, Mercer University School of Medicine, Macon, GA, USA; ^2^Atrium Health (Navicent), Macon, GA, USA; ^3^Pulmonary and Critical Care Department, Atrium Health (Navicent), Macon, GA, USA

## Abstract

Diffuse alveolar hemorrhage (DAH) is described as the collection of blood in alveolar spaces caused by damaged pulmonary vasculature. It often presents as a life-threatening medical emergency that requires urgent medical intervention along with timely diagnosis and management of the underlying cause. We hereby report a 19-year-old female who presented with clinical and radiological characteristics consistent with DAH. Laboratory workup studies revealed a diagnosis of systemic lupus erythematosus (SLE) as well as *Mycobacterium tuberculosis* (MTB) infection. This report describes an extremely unusual case of undiagnosed SLE and coexistent tuberculosis presenting as DAH. This leads to an interesting possibility of risks in patients with immune-mediated vasculitis towards developing severe pulmonary disease in the setting of pulmonary mycobacterial infection.

## 1. Case

**Figure 1:**
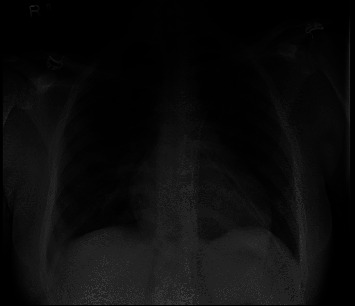
Antero-posterior (AP) view chest X-ray showing bilateral infiltrates.

A 19-year-old female sought medical attention at the primary care clinic for dyspnea on exertion (DOE) and a cough productive of blood-tinged sputum. The patient was diagnosed with pneumonia and was prescribed oral antibiotics as an outpatient. Approximately two weeks later, her hemoptysis progressed to daily episodes of 1-2 teaspoons of fresh blood and her DOE also increased to a point where she was feeling tired while performing her activities of daily living (ADL). She was then admitted to the local emergency department for further workup. Her associated symptoms included weight loss of 30 lbs, subjective low-grade nocturnal fever and drenching night sweats. She also had a past history of menorrhagia and her social history included incarceration for a year approximately 6 months ago. She denied any history of smoking, multiple sexual partners, or drug abuse. Blood workup at the hospital indicated severe anemia with hemoglobin (Hb) of 4.6 g/dL and the chest X-ray showed bilateral infiltrates. With her history of menorrhagia, she was diagnosed with chronic blood loss anemia, transfused packed red blood cells, and advised to follow-up with gynecologist. The chest X-ray was interpreted as bacterial pneumonia and the patient was discharged with oral antibiotics to follow-up in an outpatient clinic. Approximately one week later, her symptoms worsened to severe dyspnea at rest along with substernal chest pain and persistent hemoptysis and she was eventually brought to the emergency department at our hospital. On arrival, her vital signs were significant with temperature of 36.5°C, blood pressure of 126/84 mmHg, pulse 115 beats/minute, respiratory rate of 15 breaths/minute, and oxygen saturations of 90% on room air corrected to 96% with a 2-liter nasal cannula. Upon physical examination; the patient was anxious; coarse crackles were heard in bilateral lung fields; tachycardia with a regular rhythm; and 1+ pitting edema in bilateral lower extremities were noted. Initial laboratory work was notable for hemoglobin (Hbg) 8.5 g/dl, hematocrit (Hct) 28.6%, mean corpuscular volume (MCV) 91 fl, platelets 198 × 10^3^ *μ*l, serum sodium 140 mEq/L, potassium 3.5 mEq/L, chloride 110 mEq/L, bicarbonate 21 mEq/L, serum creatinine 0.66 mg/dl, blood urea nitrogen (BUN) 5 mg/dl, blood glucose 83 mg/dl, prothrombin time (PT) 14.2 second, partial thromboplastin time of 24 seconds, international normalization ratio of 1.1 and a negative respiratory viral panel. Urine analysis was remarkable for hematuria (176 RBCs/HPF) without red blood cell cast, 7 WBCs/HPF, 100 mg/dl proteinuria, and the absence of glucose or ketones. Chest X-ray revealed patchy bilateral infiltrates, as shown in Figure 1.

**Figure 2:**
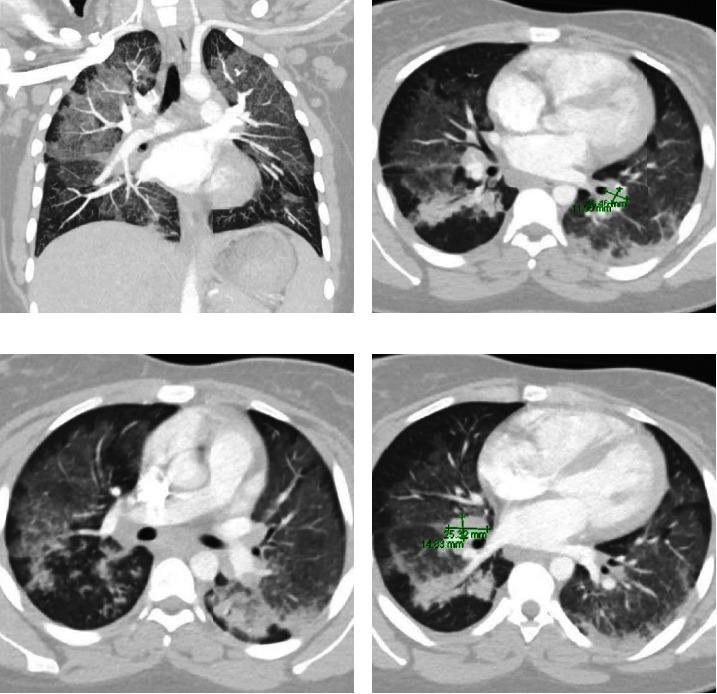
Computed topography (CT) scan showing diffuse ground glass opacities (a–d). CT scan also shows mediastinal lymph node enlargement (b, d).

The patient also underwent computed tomography (CT) of the chest with a contrast pulmonary embolism protocol, which revealed diffuse bilateral pulmonary infiltrates with ground glass and diffuse mediastinal lymphadenopathy (Figures 2(a)–2(d)).

**Figure 3:**
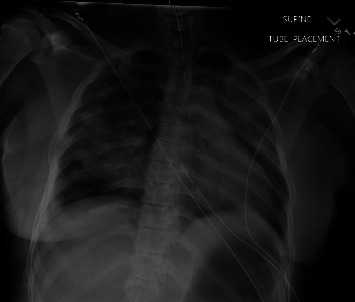
Postintubation chest X-ray showing worsening infiltrates bilaterally.

Based on the clinical and radiological findings, the patient was diagnosed with pneumonia and admitted to the medicine floor. She was started on intravenous (IV) azithromycin and ceftriaxone. However, her hypoxemia worsened in the next 12 hours with an increased oxygen requirement to 5-liter nasal cannula. Pulmonology was consulted, and the patient was moved to the intensive care unit (ICU). Given the high index of suspicion for alveolar hemorrhage, her heparin drip was stopped. Patient's oxygen requirements continued to worsen, and she was eventually intubated. Follow-up chest X-ray is shown in Figure 3.

To establish a diagnosis, flexible bronchoscopy was performed, which revealed a gross bloody return with gradual darkening on three subsequent bronchoalveolar lavages (BAL) consistent with diffuse alveolar hemorrhage (DAH). The BAL cell count was remarkable for 743,000 mm^3^ RBCs and a total WBC count of 3233 mm^3^ with 78% neutrophils and 20% lymphocytes. BAL cultures were reported negative for bacterial growth. Autoimmune workup revealed positive antinuclear antibody (ANA), high antidouble stranded DNA titer of 48 IU/ml, high antismith antibodies titers of 4.3 AU/ml, normal rheumatoid factor titers (<20 *μ*/ml), negative antibasement membrane antibody (anti-GBM), negative cytoplasmic antinuclear antibody (cANCA) and negative perinuclear antinuclear antibody (pANCA). A diagnosis of diffuse alveolar hemorrhage secondary to systemic lupus erythematosus (SLE) was made. Patient was started on pulse dose of Methylprednisolone, 1 gm IV for 5 days oral mycophenolate mofetil (MMF) for maintenance immunosuppression therapy. She also underwent 3 sessions of plasmapheresis. Her condition improved in a week, she was extubated to nasal cannula and subsequently discharged home on stable doses of prednisone and MMF. Six weeks later, the laboratory reported that her BAL cultures grew *Mycobacterium tuberculosis* (MTB). The patient was informed about the recent finding of tuberculosis and was setup in outpatient TB clinic for antituberculous therapy (ATT). She is currently finishing her 6 months of ATT with reduced dosing of MMF with close monitoring without any further complications in her condition.

## 2. Discussion

Pulmonary capillaritis comprises of a diverse group of disorders that are histopathologically characterized by the destruction and inflammation of the alveolar-capillary interface. The most common clinical manifestation of pulmonary capillaritis is DAH [1, 2]. While multiple disorders can present with DAH, all share a common denominator, which is the collection of blood in alveolar spaces due to injury to alveolar microcirculation. Causes of pulmonary capillaritis presenting as DAH can be widely separated into immune-mediated and nonimmune disorders, amongst which autoimmune vasculitis are the most frequently reported underlying condition [3–5]. DAH can be suspected based on the presence of hemoptysis, anemia, and diffuse alveolar infiltrates on radiological studies. However, DAH has a highly variable presentation, and a possibility of DAH must be considered in patients with otherwise unexplained alveolar infiltrates [2]. The management of DAH depends on the identification and treatment of the underlying cause and requires a detailed history and examination along with comprehensive laboratory studies, which must include a complete blood count with differential, blood and sputum cultures, a complete metabolic panel, an autoimmune panel including pANCA testing, antiglomerular basement membrane antibodies, antinuclear antibodies, antidouble stranded DNA, anticyclic citrullinated peptide antibodies, rheumatoid factor, antiphospholipid antibodies, creatine kinase, and urinalysis with urinary sediment, and urine drug screen [4–6]. The preferred method to confirm the diagnosis of DAH is bronchoalveolar lavage (BAL), which will show a persistent bloody return upon serial aspirations. DAH radiographically presents as patchy or diffuse, bilateral alveolar infiltrates on chest X-ray. CT is nonspecific and often shows ground glass attenuation and patchy consolidation. The histopathology of lung tissue in cases of DAH due to pulmonary capillaritis would reveal the presence of red blood cells (RBCs), fibrin, infiltrating neutrophils, or hemosiderin-laden macrophages in the alveolar spaces, fibrinoid necrosis of alveolar and capillary walls, and/or interstitial thickening [1]. In our case, the clinical syndrome of SLE was confounded by an underlying mycobacterial infection which can be a co-contributor towards the detrimental consequence of DAH.

DAH has been documented as a rare presentation in patients with SLE, with an estimated 4% of patients with SLE developing this pulmonary complication [5, 6]. The majority of cases of DAH secondary to SLE occur in young women, and in approximately 20% of cases; SLE is diagnosed after the occurrence of DAH in a patient [5]. In all such patients with SLE that present with DAH, high-dose pulse methylprednisolone remains the initial mainstay of therapy. In steroid resistant DAH, treatment may be further escalated with the use of other immunosuppressants such as azathioprine, cyclophosphamide, or IV gamma globulin. Literature suggests the use of steroids and cyclophosphamide as the most effective combination [7]. In addition to immunosuppressive therapy, plasmapheresis has been documented as a successful regimen for resolution of DAH in patients with small-vessel vasculitis [4].

DAH secondary to microbial pathogens is exceedingly rare, even more so with *Mycobacterium tuberculosis* infections. Upon literature review, DAH secondary to an infectious agent was observed in immunocompetent as well as immunosuppressed individuals, with a different profile of causative agents in each case [8]. A case of DAH secondary to miliary MTB has been reported by Nakamura et al., in which the patient quickly progressed towards respiratory failure and death, and autopsy findings and BAL confirmed the presence of *M. tuberculosis* infection [9]. A similar report of an immunocompromised patient has been presented by Saraya et al., where the patient developed DAH during a hospital stay and BAL cultures confirmed the presence of acid-fast staining mycobacterium and the diagnosis of miliary tuberculosis [10]. Furthermore, MTB presenting as DAH has also been reported with other underlying autoimmune disorders, such as ankylosing spondylitis [11]. Another case of MTB has been reported in an immunocompetent male who developed DAH, and due to high clinical suspicion, ATT was initiated as a primary therapy with rapid improvement in symptoms [12]. On the contrary, in MTB-endemic areas, other causes of DAH have been misdiagnosed as MTB-induced pulmonary disease [13, 14]. With our patient, MTB was on the list of differential diagnoses due to recurrent nocturnal fevers, weight loss, hemoptysis, and recent incarceration. Incarceration is a known risk factor for acquiring MTB, and we believe she contracted MTB during her imprisonment [15, 16]. Due to the low incidence of MTB causing DAH in immunocompetent individuals, pending results for BAL cultures, positive autoantibody titers for SLE, and a higher incidence of DAH in autoimmune vasculitis, SLE was deemed to be the most likely source of her clinical presentation. Therefore, appropriate immunosuppressant therapy was initiated based on the available laboratory data [17].

Although our patient, who was later confirmed to have pulmonary MTB, was treated with pulse dose steroids, plasmapheresis, and mycophenolate mofetil, she never decompensated from MTB infection. In fact, a literature review on the use of plasmapheresis for MTB indicates that plasmapheresis improves organ recovery in MTB and promotes disease remission [17, 18]. Moreover, studies evaluating the use of steroids in the treatment of MTB present improved outcomes with initial short-term use [19]. While it is a known fact that latent MTB can be reactivated through the long-term use of immunosuppressive agents, studies also support the use of pulse-dose steroids and plasmapheresis in improving clinical outcomes without placing the patient at great risk of reactivation.

SLE represents a syndrome with a wide spectrum of primary immune dysregulation in humoral as well as cellular components of the innate and adaptive arms of the immune system [20]. Moreover, the process of microbial infection supports the breakdown of immunological tolerance towards host antigens, further augmenting immune dysfunction [21, 22]. The causative link between infections and autoimmunity has been well studied. Combined clinical data and results indicate that infectious agents play a significant role in the induction, progression, or exacerbation of SLE [21, 23–25]. We believe that, in our case as well, active MTB infection was a significant trigger in the development and exacerbation of SLE. Literature also suggests a very complex relationship between SLE and MTB, wherein patients with SLE seem to be at higher risk of acquiring MTB infection [23, 26, 27]. Few cases of pulmonary as well as extrapulmonary tuberculosis with co-existing SLE have been reported, more so from areas where MTB is endemic [28, 29]. These studies indicate that there is a higher likelihood of procuring active MTB infection with underlying SLE [30]. While SLE patients are intrinsically immunocompromised, treatment with immunosuppressive drugs further puts them at risk of acquiring MTB infections [31, 32]. Infectious agents are also known to participate in escalating the pathogenesis of an underlying disease. Similar data has also been found regarding MTB and SLE co-existence, where MTB is implicated to exacerbate the disease and cause episodes of SLE flare [27].

## 3. Summary

Although DAH as a manifestation of MTB is uncommonly reported, it is critical that it remain high on the differential when treating patients with hemoptysis, especially if the patient has risk factors such as an underlying immune vasculitis or symptoms suggestive of MTB. We recommend the routine practice of sending BAL specimens for microbial cultures as well as molecular (multiplex PCR, MALDI-TOF MS) studies to avoid missing the possibility of an underlying infection as the trigger for induction or exacerbation of an immune-mediated disorder. The mortality rate for DAH is substantially high; however, early and aggressive treatment can improve patient outcomes. While DAH in SLE may or may not involve vasculitis, in our case we postulate that DAH was primarily due to SLE-induced alveolar capillaritis. The patient was effectively managed with immunosuppressants following presentation and continued to be in good health six to seven weeks later, when the diagnosis of MTB was revealed and ATT was initiated. Clinical studies support the early use of steroids and immunotherapy in early MTB infections, which worked in favor of our patient as she switched to ATT after resolution of DAH and SLE symptoms.

## Data Availability

The clinical case report data used to support the findings of this study are included within the article.
